# Effect of Traditional and Non-Traditionally Processed Blue Corn Tortilla Consumption During the Gestation of Rats in the Dentate Gyrus of Pups

**DOI:** 10.3390/foods13223639

**Published:** 2024-11-15

**Authors:** Paola Fernanda González-Nieto, Mayvi Alvarado-Olivarez, Rosa Isela Guzmán-Gerónimo, Juan Francisco Rodríguez-Landa, Laura Teresa Hernández-Salazar

**Affiliations:** 1 Instituto de Neuroetología, Universidad Veracruzana, Xalapa 91190, Mexico; pglez5@hotmail.com (P.F.G.-N.); juarodriguez@uv.mx (J.F.R.-L.); terehernandez@uv.mx (L.T.H.-S.); 2 Instituto de Ciencias Básicas, Universidad Veracruzana, Xalapa 91190, Mexico

**Keywords:** blue tortilla, microwave, bioactive compounds, dentate gyrus, cell density

## Abstract

The effect of consuming traditionally and non-traditionally processed blue corn tortillas on the dentate gyrus of rat pups during gestation was evaluated. Blue corn tortillas were made from grains steeped or not steeped in a solution of gallic acid and processed by traditional or microwave nixtamalization. Total polyphenol and total anthocyanin contents were analyzed. At day 20 of gestation, the pups were analyzed according to the diet administered to the pregnant rats, as follows: the control group fed with standard diet; the TN group = standard diet + blue corn tortilla by traditional nixtamalization; the TNGA group = standard diet + blue corn tortilla by traditional nixtamalization + gallic acid; the MN group = standard diet + blue corn tortilla by microwave nixtamalization; and the MNGA group = standard diet + blue corn tortilla by microwave nixtamalization + gallic acid. The cell density and soma size of the dentate gyrus in pups, along with the number of pups per litter and the litter weight, were recorded. The highest polyphenol and anthocyanin content were found in blue corn tortillas made from grains steeped in gallic acid and processed by microwave nixtamalization. The MNGA group showed larger litters as well as higher cell density (33%) and soma size (50% in the range of 30–50 μm^2^) in the dentate gyrus of pups as compared to the control.

## 1. Introduction

It is well known that nutrition plays a vital role in every stage of human life, particularly during pregnancy, where the supply of essential nutrients directly affects the growth and development of the fetus, including the structure and function of organs such as the brain, the first organ to develop. The hippocampus is one of the most important brain structures within the limbic system, which is specifically responsible for learning, memory, spatial perception, and neurogenesis [1]. The human hippocampus begins developing in the third week of gestation, with the dentate gyrus standing out as a key input area for information from other cortical areas and playing a central role in neurogenesis. The dentate gyrus contributes to the formation of new episodic memories, spontaneous exploration of novel environments, and other functions. However, abnormalities in the dentate gyrus could be responsible for more than 40% of deaths related to sudden infant death syndrome [2]. Other studies suggest that deficits in dentate granule cells (DGCs)—such as the loss of DGCs or genetic mutations—contribute to the development of various psychiatric disorders, including depression and anxiety [3]. Maternal–fetal assessment parameters are relevant to consider in studies of this nature since they provide valuable information on the growth and development of the offspring, as well as physiological and nutritional changes that influence maternal weight gain since proper nutrition during pregnancy is critical in reducing infant morbidities and mortalities.

Dietary compounds such as anthocyanins exhibit several biological properties such as antioxidant, anti-inflammatory, anti-obesity, and neuroprotective activities [4]. In a two-generation reproduction study, the administration of grape skin extract containing 225 mg anthocyanins/kg/day showed no effects on pup viability [5]. A recent study demonstrated that the administration of blackberry juice to pregnant *Wistar* rats increased the cell density in the dentate gyrus of their offspring, an effect attributed to the anthocyanin content of the juice [6]. Blue maize is also a source of anthocyanins. A study by our research group showed that grains from the Mixteco race blue maize contain mainly acylated anthocyanins [7]. The grains of blue maize are used to make tortillas, which are consumed in Mexico and by Mexican communities in the United States. It has been reported that the daily consumption of tortillas per person in rural and urban areas in Mexico is 217.9 g and 155.4 g, respectively [8].

Anthocyanins are degraded during the traditional preparation of blue corn tortillas, which involves a thermal–alkaline treatment known as nixtamalization, a process commonly used in tortilla production [9]. Previous patented work by our research group showed that the profile of anthocyanins of blue corn tortillas made with grains from Mixteco maize includes cyanidin-3-glucoside and cyanidin-3,5-diglucoside, acylated anthocyanins, as well as protoanthocyanidins [10]. On the other hand, some studies have reported some favorable effects of blue corn and tortillas from Mixteco maize on metabolic syndrome and antiproliferative activity in several cancer line cells [11,12]. Another biological study of animals observed the neuroprotective properties of blue corn tortillas made with grains from Mixteco maize by the traditional process [13]. In addition, it has been reported that cyanidin-3-glucoside possesses several beneficial biological activities in the brain [14]. However, there is limited information regarding the biological effects of blue corn tortillas on the brains of the offspring.

Given the above, blue corn tortillas from Mixteco maize may have a potential application in maternal nutrition. However, an important key factor in the development of food products rich in anthocyanins is the stability of these compounds. Several non-traditional methods for producing blue corn tortillas have been reported, including replacing calcium hydroxide with other calcium salts [15], as well as employing alternative nixtamalization technologies such as extrusion [16] and ultrasound [17]. Alternative technologies, such as microwave-based nixtamalization, could lead to the development of blue corn tortillas with higher retention of polyphenols like anthocyanins. This can be attributed to the use of high temperatures for short periods, minimizing the degradation of these bioactive compounds [18]. Additionally, organic molecules such as gallic acid have been employed to reduce the degradation of anthocyanins [19]. The use of these polyphenols in food products has additional advantages, such as the wide availability of food-grade gallic acid and its higher absorption rates as compared to other polyphenols [20]. The importance of higher retention of anthocyanin content in foods such as blue corn tortillas is due to its potential positive impact on health.

It is crucial to generate fundamental knowledge about the effects of consuming blue corn tortillas on the development of key structures within the nervous system, such as the hippocampus, particularly to evaluate the neuroprotective capacity and the potential to stimulate neurogenesis during the gestational stage, since this is a critical stage for the nervous system development. Abnormalities in the hippocampus have been associated with conditions such as autism, attention deficit disorder, and hyperactivity. Therefore, the objective of this study was to evaluate the effect of traditional and non-traditionally processed blue corn tortilla consumption during gestation of rats in the dentate gyrus of pups.

## 2. Materials and Methods

### 2.1. Preparation of Blue Corn Tortillas by Traditional Nixtamalization

**Figure 1:**
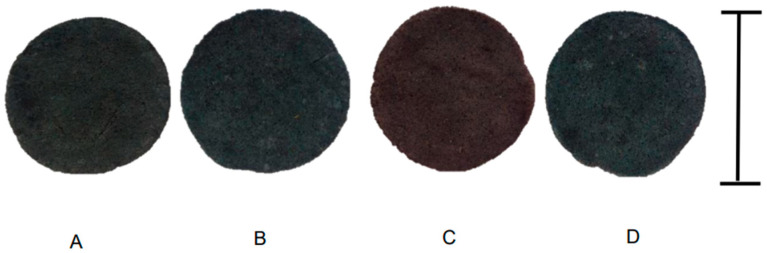
Blue corn tortillas from grains steeped in a gallic acid solution by traditional (**A**) or non-traditional process (**C**). Blue corn tortillas from grains not steeped in a gallic acid solution by traditional (**B**) or non-traditional process (**D**). Scale = 12 cm.

Grains of the Mixteco blue maize variety were collected in Chalcantongo, Oaxaca, México. Before traditional nixtamalization, a portion of the blue maize grains were steeped in a 1% solution of gallic acid for 12 h, after which the solution was removed. The grains were then cooked at 92 °C for 35 min in a 1% calcium hydroxide solution (1:2 *w*:*v*). The cooked grains of blue maize were steeped in the calcium hydroxide solution for 16 h, followed by rinsing three times with 1 L of purified water. To obtain fresh dough, 40 mL of purified water was added per 200 g of grains immediately and the mixture was ground in a manual mill. Tortillas were made by pressing the dough into disks using a domestic press and baking them on a griddle, each tortilla weighed approximately 20 g [12] (Figure 1A). Blue corn tortillas were also made from grains that had not been steeped in a gallic acid solution (Figure 1B).

### 2.2. Preparation of Blue Corn Tortilla by Non-Traditional Process

Blue maize grains, either steeped or not steeped in a 1% gallic acid solution for 12 h, were placed in a glass container with a vent to allow steam release during cooking. A 1% calcium hydroxide solution was added, and the grains were then processed in a domestic microwave oven (Panasonic, Model NN-6468, Secaucus, NJ, USA) operating at a frequency of 2450 MHz and 682 W for 7 min. Then, the grains of blue maize were steeped in the calcium hydroxide solution for 16 h and rinsed three times with 1 L of purified water. To make fresh dough, 40 mL of purified water was added per 200 g of grains, and the mixture was ground in a manual mill. A domestic press was used to make each tortilla, and the dough disks were baked on a griddle [12] (Figure 1C). Blue corn tortillas were also made from grains that had not been steeped in a gallic acid solution (Figure 1D).

### 2.3. Total Polyphenols

The Folin–Ciocalteau method was used to quantify the total polyphenol content [21]. A calibration curve was prepared using gallic acid, and the results were expressed as milligrams of gallic acid equivalents (GAE) per 100 g of sample. All determinations were performed in triplicate.

### 2.4. Total Anthocyanins

The total anthocyanin content was determined using the pH differential method [22]. The anthocyanin content was expressed as milligrams of cyanidin-3-glucoside equivalents per 100 g of sample. All determinations were performed in triplicate.

### 2.5. Experimental Animals

Recently weaned female Wistar rats were housed in acrylic cages under light/dark cycles (12/12 h, room temperature 25 °C), and given free access to a standard rodent diet (Purina Rodent Chow Lab^®^, ON, Canada) and water. All animal procedures were conducted in accordance with ethical guidelines for the proper use and housing of animals outlined in the Official Mexican Norms [23] and the Guide for the Care and Use of Laboratory Animals of the National Institute of Health [24].

The estrous cycle of each female rat was monitored through vaginal smears, and then mating was carried out. Successful copulation was confirmed by the presence of a vaginal plug and pregnancy was monitored by daily body weight measurements during the 20 days of gestation.

Blue corn tortillas were administered at a dose of 10 g/kg, starting one week before mating and during gestation. The body weight gain of pregnant rats was monitored. A total of 30 rat pups were evaluated at day 20 of gestation. The groups were distributed according to the diet administered to the pregnant rats as follows: control group (C) fed with standard diet; TN group fed with a standard diet supplemented with blue corn tortillas (6.6 mg/kg polyphenols and 2.1 mg/kg anthocyanins) prepared through traditional nixtamalization without gallic acid steeping; TNGA group fed with a standard diet supplemented with blue corn tortillas (7.99 mg/kg of polyphenols and 4.96 mg/kg of anthocyanins) prepared through traditional nixtamalization and gallic acid steeping; MT group fed with a standard diet supplemented with blue corn tortillas (9.01 mg/kg of polyphenols and 5.3 mg/kg of anthocyanins) made by microwave nixtamalization without gallic acid steeping; and MNGA group fed with a standard diet supplemented with blue corn tortillas (9.62 mg/kg of polyphenols and 6.1 mg/kg of anthocyanins) made by microwave nixtamalization and gallic acid steeping. All experimental groups received ad libitum water.

The number of pups per litter and litter weight were recorded. Male pup brains were isolated, fixed in 10% formaldehyde for 30 days, and then dehydrated using ethanol at different concentrations. After the fixing process, the brains were cut into 10 µm thick slices using a microtome (Reichert-Jung 820 II, MN, USA) and stained with Nissl stain. For analysis of cell density and cell soma size in the dentate gyrus, exclusion criteria included the null visibility of the cell nucleus. Microphotographs were taken using a visible light microscope (Leica DM750, Leica Co., Wetzlar, Germany) equipped with a camera (Lumera Infinity 1-5C, Lumera Co., Ottawa, ON, Canada). The dentate gyrus was analyzed at the level of coronal plane 11 using the atlas of the developing nervous system of the rat [25]. The cell density of the dentate gyrus was quantified in the area delimited by the dentate gyrus (cells/mm^2^), and, to determine the size of the soma, the area of each cell was considered (μm^2^) and analyzed by the ranges of 5–29 μm^2^, 30–59 μm^2^, and 60–90 μm^2^. It was measured using ImageJ v.1.5f software (National Institutes of Health, Bethesda, MD, USA).

### 2.6. Statistical Analysis

A one-way analysis of variance (ANOVA) was performed, and significant differences were evaluated using Tukey’s test at a significance level of *p* ≤ 0.05. Statistical analyses were carried out using IBM PSPP Statistics, Version 23.

## 3. Results and Discussion

Blue corn tortillas are made by the traditional nixtamalization process; therefore, the values of bioactive compounds in this study for the different blue corn tortillas were compared to those made using the traditional process. Blue corn tortillas made from grains not steeped in gallic acid solution and processed by microwave nixtamalization showed 0.35 and 1.53 times higher total polyphenol and anthocyanin content (Table 1), respectively, compared to blue corn tortillas made from grains not steeped in gallic acid solution and processed by traditional nixtamalization, a difference that was statistically significant (*p* < 0.05). The improved retention may be attributed to the application of high temperatures over a very short time during the microwave process, which minimizes the degradation of anthocyanins [18]. The statistical analysis suggests a potential application of microwaves in the nixtamalization process to enhance the anthocyanin content in blue corn tortillas, as the processing time required is shorter than the one of traditional nixtamalization.

On the other hand, blue corn tortillas made from grains steeped in gallic acid solution and processed by traditional nixtamalization showed 0.2 and 1.4 times higher total polyphenol and anthocyanin content, respectively, compared to blue corn tortillas made from grains not steeped in a gallic acid solution and processed by traditional nixtamalization (Table 1), a statistically significant difference (*p* = 0.001). Similarly, blue corn tortillas made from grains steeped in a gallic acid solution and processed by microwave nixtamalization exhibited 0.44 and 1.9 times higher total polyphenol and anthocyanin content, respectively, compared to tortillas made from grains not steeped in gallic acid and processed by traditional nixtamalization (statistically significant difference, *p* = 0.001). The statistical analysis indicates that the use of grains steeped in gallic acid solution in the elaboration of blue corn tortilla by traditional and non-traditional processes increases anthocyanin content, which are the compounds of interest in the present study due to their neuroprotective properties, as suggested by previous studies [4]. Previous studies have shown that adding gallic acid to blueberry juice, as well as Cabernet Sauvignon wine, improved anthocyanin retention, suggesting that gallic acid has a protective effect on anthocyanins [19,26]. Another study determined the effects of gallic, ferulic, and caffeic acids on the anthocyanin stability of purple sweet potatoes, where gallic acid showed the strongest protection effect. This can be attributed to the shortest distance of its aromatic ring to the anthocyanin [27]. Another factor that influences the reaction between anthocyanins and organic acids such as gallic acid is the degree of hydroxylation of the anthocyanins. For example, the cyanidin-3-glucoside molecule that has two free hydroxyl groups in the B-ring showed higher stability as compared to anthocyanins with three free hydroxyl groups in the B-ring [28]. In this research, only a quantitative analysis of anthocyanins of blue corn tortillas was included; however, a previously patented work from our research group showed that cyanidin-3-glucoside is present in blue corn tortillas made with grains from Mixteco race by traditional process [9]. Therefore, the retention of anthocyanins in blue corn tortillas made with grains steeped in a gallic acid solution by the traditional process could be attributed to the presence of cyanidin-3-glucoside. We acknowledge that qualitative analysis of anthocyanins of blue corn tortillas made by a non-traditional process should be addressed in future research.

The application of microwaves and the use of gallic acid in the nixtamalization process enhance the stability of anthocyanins, which could increase the daily polyphenol and anthocyanin intake from blue corn tortillas. The recommended daily intake of anthocyanins is 2.5 mg/kg/day [29]. Blue corn tortillas made of grains without/with steeping in gallic acid solution by traditional nixtamalization provide 0.84 and 1.98 times the daily recommended intake of anthocyanins, respectively. Meanwhile, blue corn tortillas made of grains without/with steeping in gallic acid solution and processed by microwave nixtamalization supplied 2.1 and 2.4 times the daily intake of anthocyanins, respectively.

Nutrition plays an important role throughout human life, especially during pregnancy, where nutrients play a key factor in growth and development. Since the brain is the first organ to develop, adequate nutrition is essential, and evaluating the effect of polyphenol-rich foods, such as anthocyanins, administered during pregnancy on offspring development is important.

When analyzing the effect of maternal administration of blue corn tortillas, we observed that gestational body weight gain was statistically similar across all experimental groups (Table 2). This is consistent with data reported by Ruiz-Martínez et al. [6], who administered blackberry juice to pregnant Wistar rats. Anthocyanins have been reported to suppress lipid accumulation in adipocytes through inhibition of the neuropeptide Y (NPY) and increasing γ-amino butyric acid receptor (GABAB1R) in the hypothalamus [30]. It has been reported that the cyanidin-3-glucoside found in blue corn tortillas made from grains not steeped in gallic acid solution and processed by traditional nixtamalization is a biologically active component that helps decrease weight gain [31]. In addition, a study showed that the administration of crude extract of Mixteco blue maize reduced the adipose tissue in an animal model of metabolic syndrome [11]. The anthocyanins profile of blue maize extract is constituted by acylated anthocyanins [7].

The TN group, fed blue corn tortillas made with grains not steeped in gallic acid solution and processed by traditional nixtamalization, showed the lowest number of pups per litter, although the difference was not statistically significant compared to the control group. The MNGA group exhibited the highest number of pups per litter compared to the control group, with a statistically significant difference (*p* = 0.048). This effect may be attributed to the anthocyanin content in blue corn tortillas made from grains steeped in gallic acid solution and processed by microwave nixtamalization. Anthocyanins have been reported to display phytoestrogenic activity [32], and recent studies showed that anthocyanin silver nanoparticles have a strong effect against AlCl_3_-induced infertility in rats [33].

According to Bautista et al. [34], a litter size of >11 pups is considered large, while <6 pups per litter is considered small. The TN group had the lowest litter size, consistent with the offspring of rats fed with blue corn tortillas made by traditional nixtamalization. On the other hand, the litter sizes of TNGA and MN groups were statistically similar to the control group.

Additionally, the MN group showed a litter weight statistically equivalent to the control group, while the TNGA and NMGA groups exhibited lower litter weights compared to the control group; these were statistically significant (*p* = 0.013, *p* = 0.003), and likely due to the predominance of larger litters, which distributed the weight among the pups.

**Figure 2:**
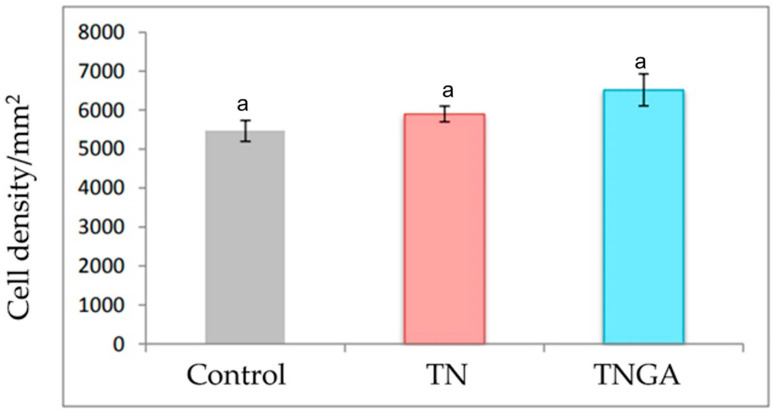
Cell density of dentate gyrus of pups from female rats fed blue corn tortillas made from grains without gallic acid solution steeping (TN) or with gallic acid solution steeping (TNGA) and processed by traditional nixtamalization. Equal letters indicate that there are no significant differences between treatments (Mean ± SE, *p* < 0.05).

In our study, we also measured cell density in the dentate gyrus of the hippocampus in the experimental groups (Figure 2). The hippocampus is a brain structure implicated in cognitive processes, essential for learning and memory, and the dentate gyrus serves as the primary input region connecting other cortical areas, playing a key role in the neural circuitry for information integration. The process of neurogenesis involves the proliferation, migration, survival, and differentiation of new cells, occurring in the dentate gyrus. This process begins during the gestation stage as part of the nervous system’s development and continues into adulthood, contributing to neuronal plasticity [35]. Pups from mothers fed blue corn tortillas made of grains without/with steeping in gallic acid solution and processed by traditional nixtamalization showed an 8% and 18% increase in cell density in the dentate gyrus compared to the control group, although the difference was not statistically significant (Figure 2).

**Figure 3:**
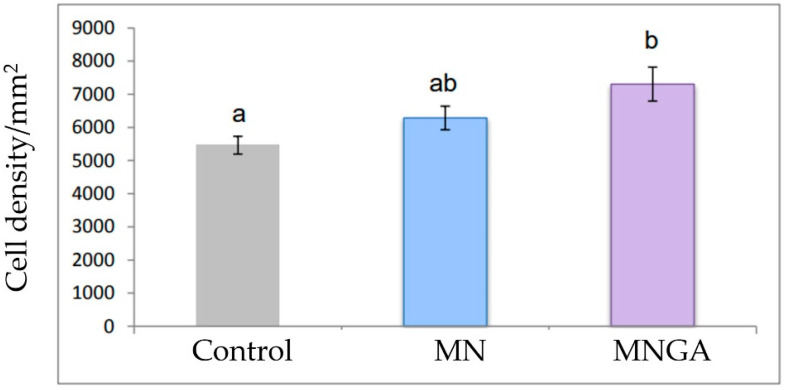
Cell density in the dentate gyrus of pups from female rats fed blue corn tortillas made from grains without gallic acid solution steeping (MN) or with gallic acid solution steeping (MNGA) and processed by microwave nixtamalization. Different letters indicate significant differences between treatments (Mean ± SE, *p* < 0.05.)

The MNGA group showed the highest cell density values among all experimental groups (Figure 3), with a 33% increase as compared to the control group, and was statistically significant (*p* = 0.013). The statistical analysis suggests that the administration of blue corn tortillas with the highest content of anthocyanins during pregnancy showed a beneficial effect on the cell density of the dentate gyrus of the offspring. So, the supplementation of blue corn tortillas made from grains steeped in gallic acid solution and processed by microwave nixtamalization at a dose of 10 g/kg supplied 6.1 mg of anthocyanins. The human equivalent dose was 1.1 mg/kg [36]. According to these data, for example, a pregnant woman of 60 kg should consume, per day, approximately 5.4 pieces of blue corn tortillas made from grains steeped in gallic acid solution and processed by microwave nixtamalization, which provides 66 mg of anthocyanins.

On the other hand, similar results were reported in the dentate gyrus of offspring from mothers administered blackberry juice [6]. Additionally, the administration of blue corn tortillas made with grains of blue maize from Mixteco race through the traditional process has been previously reported to improve the hippocampus size and the nuclear CA3 area of hippocampal neurons in mice [13].

The use of the gallic acid solution and microwave process in the production of blue corn tortillas may result in a lower degradation of anthocyanins such as cyanidn-3-glucoside, a biologically active compound with neuroprotective properties, which have been previously reported in blue corn tortillas made with grains of Mixteco blue maize and through the traditional process. However, a qualitative analysis of anthocyanins from blue corn tortillas made by traditional and non-traditional processes should be addressed in future work.

On the other hand, the increase in cell density may be related to the potential of anthocyanins to improve blood irrigation, since it is known that these compounds can enhance vascular endothelium and promote nitric oxide production, which acts as a vasodilator, increasing blood flow and nutrient amount delivery, thus favoring neurogenesis. An adequate blood supply is important for optimal neurodevelopment of the offspring and blood flow is essential for the adequate supply of nutrients, oxygen, and other substances involved in the same, which are in greater demand during the last days of gestation. This is relevant for cell differentiation and proliferation since the formation of blood vessels helps maintain neuronal growth. The increase in blood flow generated by anthocyanins may be related to the production of nitric oxide, a substance that facilitates blood flow, generating a greater availability of nutrients, oxygen, and growth factors that regulate cell proliferation and differentiation, such as those found in the dentate gyrus [37].

It has been reported that cyanidin-3-glucoside can accumulate within the brain endothelial cells [38], and this compound is present in blue corn tortillas made through the traditional process [10].

The effects of anthocyanins evaluated at adult ages in murine models have been focused on their potential to interact with proteins involved in neuronal plasticity, as well as to increase the brain-derived neurotrophic factor (BDNF), which enables them to promote learning and memory [39].

**Figure 4:**
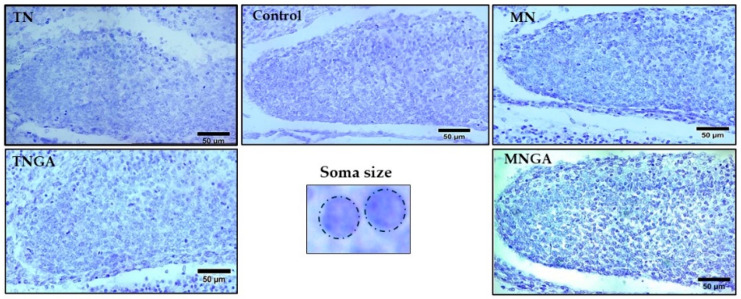
Micrographs of the dentate gyrus of pups from rats fed with standard diet (Control) and blue corn tortillas made from grains without gallic acid solution steeping (TN) or with gallic acid solution steeping (TNGA) and processed by traditional nixtamalization and blue corn tortillas made from grains without gallic acid solution steeping (MN) or with gallic acid solution steeping (MNGA) and processed by microwave nixtamalization. Scale = 50 μm.

**Figure 5:**
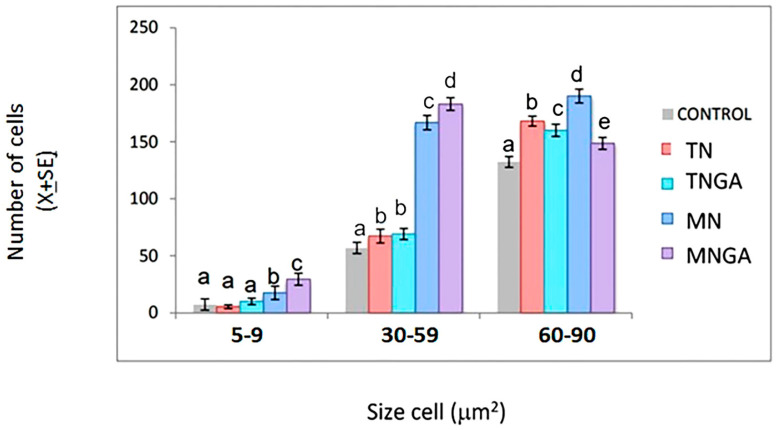
Cell soma size in the dentate gyrus of pups from mothers fed blue corn tortillas during pregnancy. Control group; TN = group fed blue corn tortillas made from grains not steeped in gallic acid solution and processed by traditional nixtamalization; TNGA = group fed blue corn tortilla made from grains steeped in gallic acid solution and processed by traditional nixtamalization; MN = group fed blue corn tortillas made from grains not steeped in gallic acid solution and processed by microwave nixtamalization; MNGA = group fed blue corn tortillas made from grains steeped in gallic acid solution and processed by microwave nixtamalization. Different letters indicate significant differences between treatments (Mean ± SE, *p* < 0.05).

The size of the cell soma can vary according to the development stage and may indicate neuronal maturity. The cells during the gestation stage are not fully developed, however, they provide insight into the cells that predominate in postnatal stages (Figure 4). Interestingly, we found that pups from mothers fed with blue corn tortillas made without/with grains steeped in gallic acid solution processed by microwave nixtamalization showed an increase in the soma size in the range of 30 to 59 μm^2^ (50%) in the dentate gyrus respect to the control group (Figure 5). These data are relevant since the information on the soma size in prenatal stages is scarce, and, therefore, these results form the basis for future studies.

The dentate gyrus is primarily composed of granular cells, which are smaller than other cell types like pyramidal cells. These cells have glutaminergic connections, essential for cognitive processes. The soma contains essential organelles for energy generation (mitochondria) and protein synthesis (ribosomes), which could be indirectly related to the production of neurotransmitters that are released during the synaptic processes, particularly in neuronal chemical transmission. Therefore, a larger soma size could support the presence of a greater number of organelles, which in turn could generate more neuronal connections.

In the ≥60 μm^2^ soma size range (60 to 90 μm^2^) (Figure 5), all experimental groups showed a high content of large cells in the dentate gyrus, especially pups in the MN group from mothers fed with blue corn tortillas made of grains processed by microwave nixtamalization, which presented a 44% and 13% increase as compared to the control group and TN group, respectively. In this study, a lower number of smaller soma cells (<30 μm^2^) is expected, as the peak of gliogenesis occurs after birth [40]. Neurological conditions such as schizophrenia and major depressive disorder have been associated with reduced neuron soma size in the hippocampus in humans [41,42].

Feeding between mother and calf is highly sensitive to maternal diet and is critical during the early stages of life. The ability of mothers to share nutrients with their offspring varies significantly, which can lead to inconsistent results in experimental studies. Nutrient intake can be associated with intrauterine growth, which makes it difficult to know the metabolic response in each individual and the inherent limitations in the generalization of the results that impact the research conducted.

During gestation, most of the connections and neuronal circuits that will serve to establish future cognitive functions are established. Therefore, having a higher cell density in the dentate gyrus may favor the establishment of more neuronal networks in adult ages, which would imply that connections to other cortical areas are related to mechanisms that involve essential processes such as memory.

## 4. Conclusions

The addition of gallic acid helps enhance anthocyanin retention in blue corn tortillas made by traditional and non-traditional processes. The application of microwaves in the nixtamalization process decreased processing time as compared to traditional nixtamalization and also increased the retention of anthocyanins. Blue corn tortillas made from grains steeped in a gallic acid solution and processed by microwave nixtamalization showed the highest content of anthocyanins; when administered during pregnancy, an increase in cell density and larger cell somas in the dentate gyrus of pups was observed. This research contributes to the exploration of traditional foods rich in anthocyanins and the application of a non-traditional process to obtain food products with health-promoting effects in the offspring. These findings highlight the importance of the dentate gyrus in the variations in histological characteristics; although the rat pup brain is not fully formed, the study of the number of cells in the dentate gyrus and the size of the soma reveal information that helps understand neuronal development as a crucial aspect in the capacity to generate new neurons throughout life. Further research is needed to know the changes in the profile of anthocyanins of blue corn tortillas made through several processes and their influence on the biological properties of this food product.

## Figures and Tables

**Table 1:** Total polyphenol and total anthocyanin content in blue corn tortillas processed by traditional and non-traditional nixtamalization.

Blue Corn Tortillas *	Total Polyphenol mg GAE/100 g	Total Anthocyanin mg C3GE/100 g
Blue corn tortilla processed by traditional nixtamalization/grains without gallic acid	66.5 ± 0.2 ^a^	20.9 ± 2.8 ^e^
Blue corn tortilla processed by microwave nixtamalization/grains without gallic acid	90.1 ± 0.4 ^b^	53.0 ± 1.4 ^f^
Blue corn tortilla processed by traditional nixtamalization/with gallic acid	79.9 ± 0.1 ^c^	49.6 ± 0.3 ^f^
Blue corn tortilla processed by microwave nixtamalization/with gallic acid	95.9 ± 0.3 ^d^	61.0 ± 1.8 ^g^

* Different letters indicate significant differences between treatments (*p* < 0.05).

**Table 2:** Gestational weight gain, number of pups per litter, and litter weight experimental groups.

	*C	TN	TNGA	MN	MNGA
Gestational weight gain (g)	53.4 ± 13.4 ^a^	45.6 ± 15.1 ^a^	53.6 ± 22.9 ^a^	64.8 ± 4.31 ^a^	63.9 ± 4.65 ^a^
Number of pups per litter	9.28 ± 0.95 ^c^	5.71 ± 2.81 ^d^	7.42 ± 3.86 ^c^	10.4 ± 0.57 ^c^	13.0 ± 0.57 ^e^
Litter weight (g)	3.74 ± 4.06 ^f^	3.17 ± 0.34 ^g^	2.98 ± 0.0 ^g^	3.29 ± 0.16 ^f^	2.86 ±0.16 ^g^

*C = control group, TN = group fed blue corn tortillas made from grains not steeped in gallic acid solution and processed by traditional nixtamalization; TNGA = group fed blue corn tortillas made from grains steeped in gallic acid solution and processed by traditional nixtamalization; MN = group fed blue corn tortilla made from grains not steeped in gallic acid solution and processed by microwave nixtamalization; MNGA = group fed blue corn tortilla made from grains steeped in gallic acid solution and processed by microwave nixtamalization. Different letters indicate significant differences between treatments (Mean ± SE, *p* < 0.05).

## Data Availability

The datasets presented in this article are not readily available because the data are part of an ongoing study. Requests to access the datasets should be directed to the corresponding authors.
